# Semantic segmentation of UAV remote sensing images based on edge feature fusing and multi-level upsampling integrated with Deeplabv3+

**DOI:** 10.1371/journal.pone.0279097

**Published:** 2023-01-20

**Authors:** Xiaolong Li, Yuyin Li, Jinquan Ai, Zhaohan Shu, Jing Xia, Yuanping Xia

**Affiliations:** 1 Faculty of Geomatics, East China University of Technology, Nanchang, Jianxi, China; 2 Key Laboratory of Mine Environmental Monitoring and Improving Around Poyang Lake, Ministry of Natural Resources, East China University of Technology, Nanchang, Jiangxi, China; 3 CNNC Engineering Research Center of 3D Geographic Information, East China University of Technology, Nanchang, Jiangxi, China; Universidade do Vale do Rio dos Sinos, BRAZIL

## Abstract

Deeplabv3+ currently is the most representative semantic segmentation model. However, Deeplabv3+ tends to ignore targets of small size and usually fails to identify precise segmentation boundaries in the UAV remote sensing image segmentation task. To handle these problems, this paper proposes a semantic segmentation algorithm of UAV remote sensing images based on edge feature fusing and multi-level upsampling integrated with Deeplabv3+ (EMNet). EMNet uses MobileNetV2 as its backbone and adds an edge detection branch in the encoder to provide edge information for semantic segmentation. In the decoder, a multi-level upsampling method is designed to retain high-level semantic information (e.g., the target’s location and boundary information). The experimental results show that the mIoU and mPA of EMNet improved over Deeplabv3+ by 7.11% and 6.93% on the dataset UAVid, and by 0.52% and 0.22% on the dataset ISPRS Vaihingen.

## 1. Introduction

Nowadays, UAV low-altitude remote sensing has become an essential technical tool for rapid national natural resources investigation [1], emergency mapping [2], and disaster monitoring [3]. However, its high spatial resolution characteristics bring about problems such as complicated feature categories, significant changes in target scale, rich texture details, and intricate contour boundaries, which bring great challenges to image segmentation [4]. Therefore, it is crucial to develop algorithms that can achieve high-precision intelligent segmentation of UAV low-altitude remote sensing images.

In the last decade, image segmentation based on deep learning (DL) has achieved promising application results. Convolutional neural networks (CNNs) [5] are the most commonly used DL models in image segmentation. Fully convolutional network (FCN) [6] achieves high segmentation accuracy on standard datasets (PASCAL VOC) by replacing the fully connected layer of CNN with a fully convolutional layer, allowing images of arbitrary size as inputs [7], and demonstrates the powerful performance of deep convolutional neural networks in semantic segmentation.

The Deeplab semantic segmentation network was improved from FCN and has been developed to Deeplabv3+ [8], which combines the advantages of encoder-decoder structure and spatial pyramid pooling (ASPP) [9] module and has shown an excellent comprehensive performance in semantic segmentation recently. Wang et al. [10] investigated the application of Deeplabv3+ in remote sensing of forest fires and achieved satisfying segmentation performance and running speed; Zhang et al. [11] performed urban land use classification based on Deeplabv3+ and optimized the classification results using the fully connected conditional random field (CRF); Wang et al. [12] integrated class feature attention mechanism into Deeplabv3+ and improved the segmentation accuracy, but it still has problems of not being able to accurately segment small targets and having numerous model parameters. The above studies show that Deeplabv3+ performs quite well in semantic segmentation of remote sensing images, but its network structure is complex and requires a lot of computational resources and time to converge during training. In addition, its large upsampling amplitude leads to severe loss of pixel information [13]. For semantic segmentation of high-resolution remote sensing images, it still has problems such as low accuracy of small target recognition and poor edge recognition.

Lightweight BiSeNetV2 [14] uses detail branching and semantic branching to balance low-level and high-level semantic information. Detail branching captures low-level detail and generates high-resolution feature representations. Semantic branching is a lightweight convolutional model that uses fast downsampling to expand the perceptual field while designing contextual embedding blocks. Although it substantially reduces the number of parameters, its segmentation accuracy is not promising.

To solve the above problems, an improved Deeplabv3+ is proposed in this paper, which uses the lightweight MobileNetV2 [15] as the backbone network, and improves the accuracy of semantic segmentation using edge features provided by edge branches. Meanwhile, the decoding part uses a multi-level upsampling to enhance the tight connection between the encoder and decoder to retain the target’s location and boundary information more completely. Experimental results on the publicly available datasets UAVid [16] and ISPRS Vaihingen [17] show that the proposed model is more effective and robust than mainstream segmentation models.

The main contributions of this work are summarized as follows:

1. A semantic segmentation algorithm for UAV remote sensing images based on improved Deeplabv3+ is proposed to effectively utilize edge features and low-level image features.
2. The edge detection network built by 6 gating mechanism modules (Gate) can effectively extract edge features to improve the segmentation performance.
3. A multi-level upsampling method is designed in the decoder to retain the target’s position and boundary information when restoring the feature map more completely.

## 2. Literature review

With the development of aviation technology, satellite remote sensing technology is favoured by researchers because of its low cost and easy access [18]. In the past few years, more and more research has been conducted using DL to process remote sensing images, such as land cover classification based on hyperspectral images [19], multi-scale geospatial target detection [20], semantic segmentation of urban scenes [21], and DL has proven to be effective in processing remote sensing images.

Aerial imaging has become a common approach to acquiring data with the advent of Unmanned Aerial Vehicles (UAV). Compared with satellite-based aerospace remote sensing, UAV remote sensing can fly at low altitudes under clouds, making up for the fact that clouds often block satellite optical remote sensing from obtaining high-quality images [22]. Manual visual detection of multiple objects in an image is a time-consuming, biased and inaccurate operation. Therefore, designing algorithms that can quickly and accurately obtain information from images of this kind is a recent major challenge. Many researchers have proposed various image segmentation methods, which can be divided into three categories: traditional methods, methods based on machine learning and methods based on DL.

For remote sensing images, traditional segmentation methods mainly include threshold segmentation algorithms and edge detection segmentation algorithms. In order to improve the real-time performance of segmentation, Cheng et al. [23] proposed a threshold segmentation algorithm based on sample space reduction and interpolation methods. Xu et al. [24] used the traditional edge detection operator to solve the two-dimensional function and then selected the corresponding threshold to extract the edges of the image to realize the segmentation of UAV remote sensing images. Traditional methods are also effective in solving image segmentation tasks when dealing with images of desirable quality.

In addition, machine learning algorithms such as K-nearest neighbrs, decision tree, random forests, and support vector machines are also used for image segmentation tasks.

Cariou et al. [25] improved K-nearest neighbor method for density-based pixel clustering of hyperspectral remote sensing images for image segmentation. Yang et al. [26] combined the image digital surface model (DSM) and texture information to extract rice fallout areas using the maximum likelihood method and a decision tree classification model. Feng et al. [27] applied random forest and texture analysis to urban vegetation mapping of UAV remote sensing; Ma et al. [28] combined random forest and support vector machine for UAV remote sensing land cover classification. Although the above methods perform well in some cases, they are usually only applicable to a small range of data and cannot be validated on large datasets due to poor generalization ability [29].

DL has been widely used in semantic segmentation tasks in recent years and has performed well. As a result, many semantic segmentation methods based on DL have been applied to remote sensing image segmentation, as shown in Table 1. Ghorbanzadeh et al. [30] used CNN for landslide detection; Yang et al. [31] used CNN to extract mature rice areas and estimate rice production automatically; Su et al. [32] improved the CNN and proposed a new rice lodging identification method; Wang et al. [21] combined convolution with transformer to achieve semantic segmentation of urban scene imagery. The Deeplab series algorithm has shown outstanding performance in semantic segmentation in recent years. Based on Deeplabv1 [33], the researchers have proposed Deeplabv2 [9], Deeplabv3 [34], and Deeplabv3+ [8], which gradually improve the algorithm segmentation performance by optimizing the network structure. Wang et al. [10] performed remote sensing of forest fires based on Deeplabv3+ and achieved quite well segmentation performance; Zhang et al. [11] achieved promising results in urban land use classification based on Deeplabv3+ and UAV remote sensing technology; Wang et al. [12] added a class feature attention mechanism to Deeplabv3+ and achieved high overall segmentation accuracy; Du et al. [35] incorporated Deeplabv3+ and object-based image analysis strategy to label remote sensing image, which achieves impressive accuracy.

**Table 1:** Summary of remote sensing image segmentation methods based on DL.

Method	Application	Reference	Contribution(s)
CNN	Regional segmentation of rice	[31]	Learn CNN directly of segmentation
	Landslide detection	[30]	Research the application of machine learning methods and different CNN models for landslide detection
FCN	Forest fire detection	[10]	Research the application of FCN models for forest fire scenarios
	Remote sensing image segmentation	[12]	Class feature attention mechanism combine with DCNN
	Cloud Detection	[36]	Channel attention module combine with lightweight DCNN
FCN + RCF	Land use classification	[11]	RCF optimizes the results of the model classification
Deep + Shallow structures	Rice collapse detection	[32]	Combine deep and shallow structures
FCN + OBIA	Automatic annotation of remote sensing images	[35]	Object-based image analysis strategy combined with DCNN
CNN + Meta-learning	Land cover classification	[37]	Meta-learning approach to learning what is learned in a deep-shallow structure
FCN + Transformer	Urban scene segmentation	[21]	Combine Transformer and DCNN

In addition, the balance between the accuracy and efficiency of detection models in large-scale remote sensing image segmentation tasks is also a research point of interest. Yao et al. [36] combined the channel attention mechanism with a lightweight deep convolutional neural networks (DCNN) to achieve efficient cloud detection on remote sensing images. For the convenience of readers, we summarize the above methods in Table 1. The above studies have improved Deeplabv3+ make it more suitable for remote sensing image semantic segmentation tasks, but there is still room for improvement in edge fineness and small target recognition accuracy.

## 3. Methodology

Currently, Deeplabv3+ is a well-performing deep semantic segmentation model that uses the ASPP module and the encoder-decoder structure. The former captures multi-scale contextual information by pooling feature layers at different resolutions and the latter capturing clearer object boundaries. In ASPP, multi-scale features are captured by parallel null convolution with different expansion rates. Then the stitched feature maps are fed into a 1×1 convolutional layer, and the output feature maps are used as the output of the encoder. In the decoding part, the feature maps output by the encoder is first 4-fold bilinearly upsampled and then connected with the corresponding size low-level feature maps extracted from Xception [38] backbone network. In this case, another 1×1 convolution is used for the low-level features to reduce the number of channels in network layers. After joining, the features are refined using 3×3 convolution, and then 4-fold bilinear upsampling is performed again to ensure that the output segmentation map is as large as the original image.

However, during the downsampling of the feature map by the encoder, as the number of layers in the network deepens, the resolution of the feature map gradually decreases, and the features of small targets are gradually blurred. At the same time, the null convolution with a significant void rate in ASPP is not conducive to segmenting low-resolution feature maps [39]. In the upsampling phase of the feature map, the decoder part does not fully use the multi-level feature map generates by the encoder and directly quadruples the bilinear upsampling of the feature map, which is not conducive to pixel-level information.

**Fig 1:**
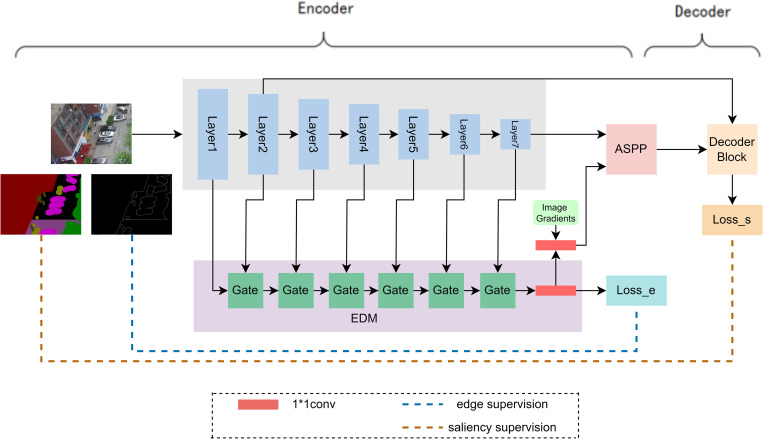
EMNet structure.

To solve the above problems, an improved EMNet based on Deeplabv3+ is proposed. As shown in Fig 1, EMNet mainly consists of an encoder and a decoder, and the encoder contains a semantic segmentation module and an edge detection module.

### 3.1. Semantic segmentation module

As shown in Fig 1, the semantic segmentation module consists of a backbone feature extraction network and an ASPP module. In order to reduce the model computation and memory footprint so that image features can be mined more efficiently and quickly [36], EMNet uses the lightweight MobileNetV2 network as the backbone feature extraction network. Compared with the Xception network of Deeplabv3+, this network has shallower layers, fewer parameters, lower model complexity, and faster convergence. The structure of MobileNetV2 network is shown in Table 2, where t is the multiplication factor (i.e., expansion factor) of the input channels, c denotes the number of output channels, n represents the number of repetitions of the module, while s is the step size.

**Table 2:** Structure diagram of MobileNetV2 network.

Input	Operator	t	c	n	s
12*512*3	conv2d	-	32	1	2
56*256*32	bottleneck	1	16	1	1
28*128*16	bottleneck	6	24	2	2
4*64*24	bottleneck	6	32	3	2
2*32*32	bottleneck	6	64	4	2
2*32*64	bottleneck	6	96	3	1
2*32*96	bottleneck	6	160	3	2
2*32*160	bottleneck	6	320	1	1

### 3.2. Edge detection module

Deeplabv3+ captures the colour, shape, and texture information together using DCNN, which reduces the segmentation accuracy due to the aggregation of all the different types of information related to the recognition target at the bottom layer of the network. In comparison, the edge detection branch of EMNet can capture and learn the edge features of the input image solely, which helps to obtain more detailed information and thus can provide adequate edge information for semantic segmentation.

**Fig 2:**
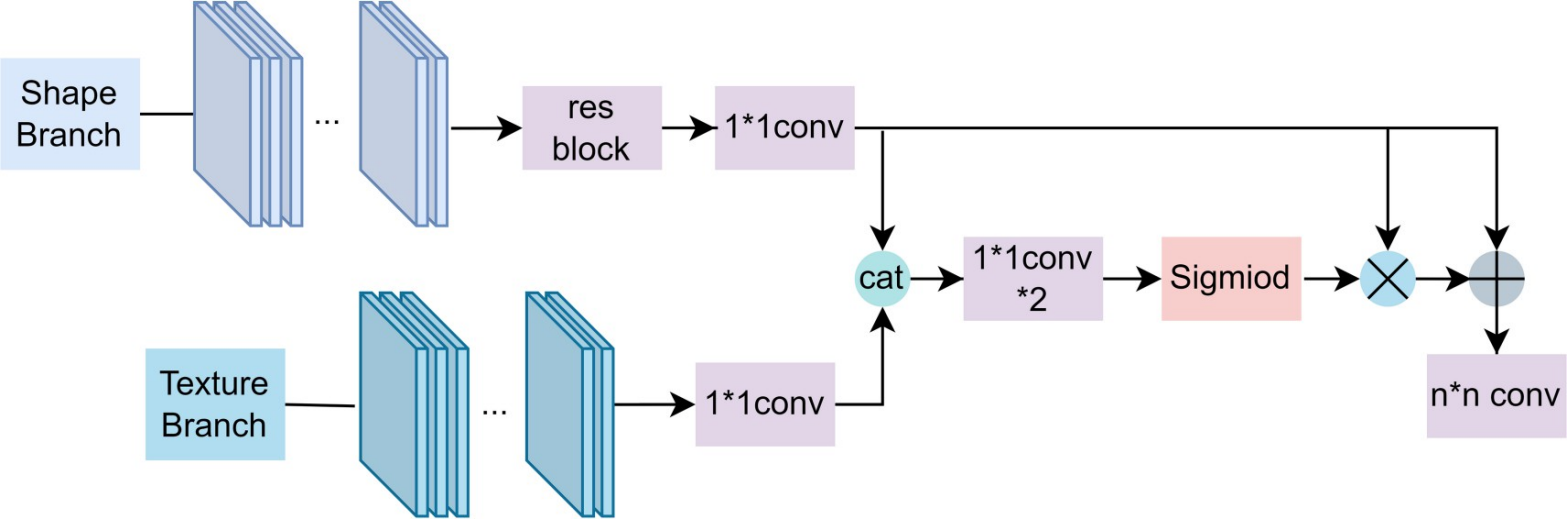
Gate structure.

Edge detection module (EDM) takes the output of each layer of the Mobilenetv2 network as its input. Borrowing from the literature [40], EDM module is designed to consist of six gating mechanism modules (Gate), and the specific structure of the Gate is shown in **Fig 2**.

**S**_*t*_ denotes the edge stream, **T**_*t*_ denotes the semantic stream, || denotes the connection of feature mapping, *C* denotes convolutional operation, and **α**_*t*_ can be considered as an attention graph that assigns greater weight to regions with important boundary information. The Gate first uses a residual block and a 1×1 convolutional block to extract, downsample and upsample the input edge feature stream **S**_*t*_, and downscales the input semantic stream **T**_*t*_ using a 1×1 convolutional block. Then the features of these two streams are fused, and the output feature map is reduced in dimension using two 1×1 convolutional blocks. And finally, we use the sigmoid function *S* to restrict the output to the range of [0, 1] so that each value in the output vector can represent the weight of its corresponding channel feature in the input feature as implemented in Eq (1).


$$\alpha_{t}=S(C_{1\times 1}(S_{t}||T_{t}))$$ (1)

**V**_*t*_ denotes the edge stream **S**_*t*_ processed by the residuals and the 1×1 convolutional block, and then **V**_*t*_ and **α**_*t*_ are connected by residuals. Finally, channel-wise weighting with kernel *w*_*t*_ to obtain a feature map ${\hat{S}}_{t}$ with prominent edges:

$${\hat{S}}_{t}^{\left(i,j\right)}={\left((V_{t_{\left(i,j\right)}}⊙\alpha_{t_{\left(i,j\right)}})+V_{t_{\left(i,j\right)}}\right)}^{T}w_{t}$$ (2)

The edge feature map obtained by EDM module is upsampled back to the input image size after channel downsampling on the one hand. Then the edge extraction process is supervised using the edge labels transformed from semantic segmentation labels. On the other hand, the edge feature map is transferred to the ASPP module and fused with advanced semantic features to provide edge information for semantic segmentation. Moreover, as shown in Fig 1, we first use the Canny edge detection operator to obtain the edges of semantic segmentation labelled images, then take the edges as the image gradient, which will be fused with the edge features outputted from EDM, and finally transfer the fused features to the ASPP module. This enhances the edge weight of the feature map and thus solves the problem of edge information loss due to downsampling during feature extraction.

### 3.3. Decoder module

During the gradual downsampling of the image in encoding, the boundary information of the target is gradually blurred, and after the upsampling of the feature map by the decoder, the edges of the target are even more blurred, resulting in poor segmentation performance. Compared with satellite remote sensing images, higher accuracy of boundary contour extraction is required when semantic segmentation is performed on UAV remote sensing images.

**Fig 3:**
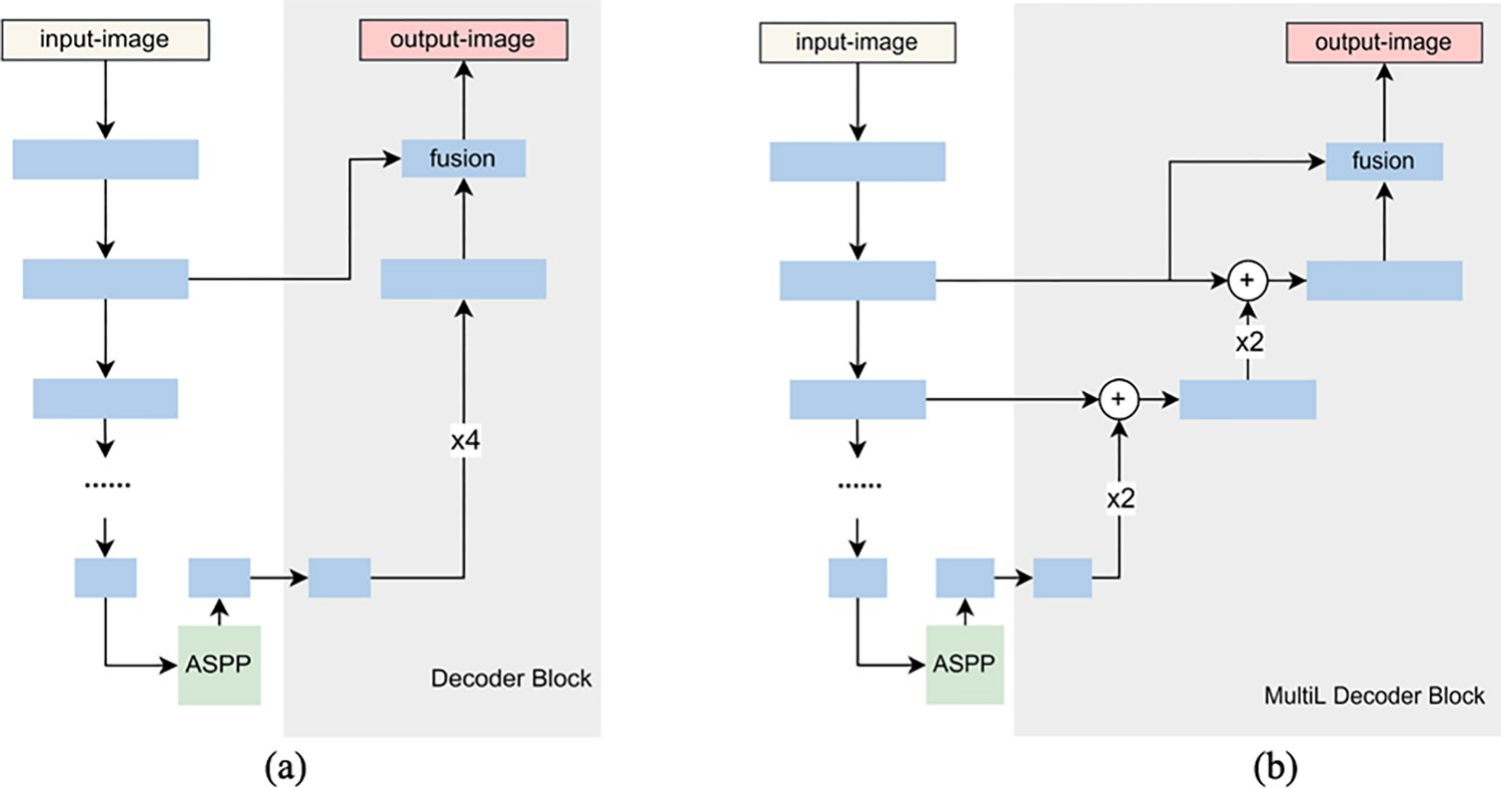
Comparison of the decoder block of Deeplabv3+ and EMNet. (a) Deeplabv3+ decoding module per-forms 4-fold bilinear upsampling; (b) EMNet decoding module performs multi-level upsampling.

In Fig 3(A), Deeplabv3+ recovers the feature maps directly by 4-fold upsampling for advanced semantic features in the decoding process. This decoding method has promising performance when applied to satellite remote sensing images, but will lose much detailed information for UAV remote sensing images, which makes the network’s segmentation performance not good enough. Considering that in the encoder, the input images are also gradually transformed from low-level to high-level semantic features through feature extraction. By supplementing the low-level feature of the corresponding size of the encoder module with the high-level semantic features of decoder for feature fusion, it is possible to compensate for some of the location and boundary information of the target lost in the process of recovering the feature map. Therefore, EMNet in this paper is designed to integrate a multi-level upsampling module (Multi-level, MultiL).

As shown in Figs 1 and 3(B), the information obtained from EDM module is transferred to the ASPP module, which is fused with the high-level semantic features outputted from the semantic segmentation backbone network to provide edge information for the semantic segmentation task. The advanced semantic features outputted by the ASPP module are recovered from the feature map by using 2-fold upsampling twice. After each upsampling operation, the semantic features are summed with the feature map of the same size in the encoding, enhancing the tight connection between the encoder and decoder. The number of channels remains unchanged after the connection summation, while the number of parameters is reduced. At the same time, the location and boundary information of the target can be retained more thoroughly.

MultiL can be described as Eq (3), where *U* denotes the upsampling operation, *k* denotes the upsampling multiplier, and *C* denotes the convolutional operation. The feature layer **D**_*i*_ in the decoder is first upsampled. Then the feature map **E**_*i*_ of the corresponding size in the encoder is up-dimensioned by 1×1 convolution and added with **D**_*i*_ to perform the feature fusion.


$$F_{fusion}=U^{k}(D_{i})+C_{1\times 1}(E_{i})$$ (3)

### 3.4. Loss function

Inspired by the idea of multi-task learning, we combine the prediction losses of semantic segmentation and edge detection modules as the final loss:

$$L_{f}=L_{S}+\rho L_{e}$$ (4)

where *L*_*S*_ is the loss of the semantic segmentation task, *L*_*e*_ denotes the loss of the edge detection task, and *ρ* represents the weight of the loss of the edge detection task.

A multi-class cross-entropy function is used to calculate the loss for the semantic segmentation task. As shown in Eq (5), *N* denotes the number of pixels, *L*_*S*_ denotes the loss of all pixels, and *l*_*spixel*_ is the loss of a single pixel.


$$L_{s}=\sum_{i=1}^{N}l_{spixel}^{i}$$ (5)

*l*_*spixel*_ can be calculated as:

$$l_{spixel}=-\sum_{k=1}^{C}y_{\left(i,j\right)}^{k}logP_{\left(i,j\right)}^{k}$$ (6)

where *C* is the number of predicted categories, $y_{\left(i,j\right)}^{k}$ is the true label of the pixel at location (*i*, *j*), $P_{\left(i,j\right)}^{k}$ is the predicted probability of the corresponding category *k* at location (*i*, *j*).

The task loss of the edge detection branch is calculated using a binary cross-entropy function. As shown in Eq (7), *N* is the number of pixels, and *L*_*e*_ denotes the loss of all pixels, *l*_*epixel*_ denotes the loss of a single pixel

$$L_{e}=\sum_{i=1}^{N}l_{epixel}^{i}$$ (7)

*l*_*epixel*_ can be calculated as

$$l_{epixel}=-[y_{\left(i,j\right)}\alpha_{1}logP_{\left(i,j\right)}+(1-y_{\left(i,j\right)})\alpha_{2}log({1-P}_{\left(i,j\right)})]$$ (8)

where *y*_(*i*,*j*)_ = {0,1}, which denotes the true label of the pixel at (*i*, *j*) position, *P*_(*i*,*j*)_∈(0,1), which denotes the predicted probability of the positive label at (*i*, *j*) position, *α*_1_ and *α*_2_ denote the weights of labels:

$$\alpha_{1}=\lambda ∙\frac{\left|Y^{+}\right|}{\left|Y^{+}|+|Y^{-}\right|}$$ (9)

$$\alpha_{2}=\frac{\left|Y^{-}\right|}{\left|Y^{+}|+|Y^{-}\right|}$$ (10)

where |*Y*^+^| denotes the number of pixel points at the edge in the image, |*Y*^−^| indicates the number of pixel points at the non-edge in the image. Considering that the number of edge pixels in the edge detection task is small, inspired by Liu et al. [41], we use *λ* to adjust the weight of positive labels.

### 3.5. Evaluation metrics

UAV remote sensing image segmentation is a sub-task of semantic segmentation, so we can directly adopt the evaluation criteria commonly used in semantic segmentation: Mean Pixel Accuracy (mPA) and Mean Intersection Over Union (mIoU). PA is mainly used to evaluate pixel-level classification accuracy for each category. mPA is averaged over all categories. IoU is used to evaluate the segmentation effectiveness of models for each category separately. mIoU is averaged over all categories. Higher values of mPA and mIoU represent better segmentation overall performance of models. For each category i, *TP*^*i*^ represents the number of pixels predicted to be true for positive samples; *FP*^*i*^ represents the number of pixels predicted to be false for positive samples; *TN*^*i*^ represents the number of pixels predicted to be true for negative samples, *FN*^*i*^ represents the number of pixels predicted to be false for negative samples, and k is the number of segmentation categories

$$mPA=\frac{1}{k}\sum_{i=0}^{k}\frac{{TP}^{i}+{TN}^{i}}{{FP}^{i}+{FN}^{i}+{TP}^{i}+{TN}^{i}}$$ (11)

$$mIoU=\frac{1}{k}\sum_{i=0}^{k}\frac{{TP}^{i}}{{FP}^{i}+{FN}^{i}+{TP}^{i}}$$ (12)

## 4. Experimental evaluation

### 4.1. Dataset

The models are trained and tested on two publicly available datasets: the UAVid dataset and the ISPRS Vaihingen dataset.

**Fig 4:**
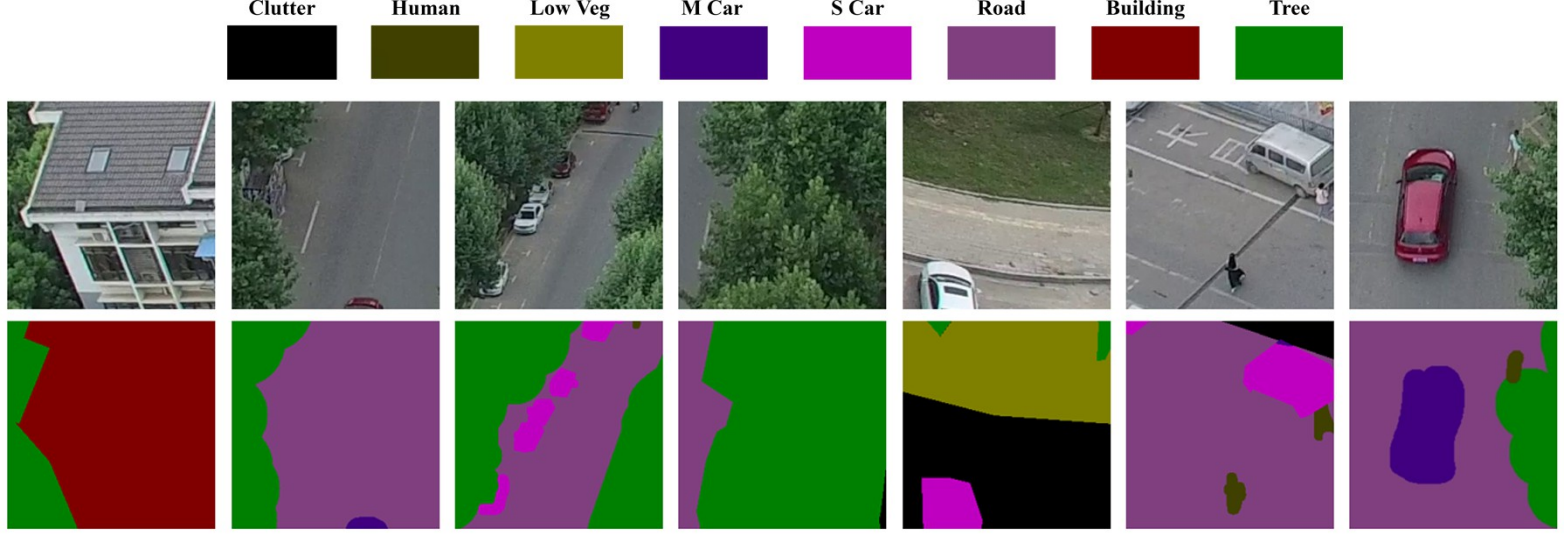
UAVid dataset example. Reprinted from [16] under a CC BY license, with permission from [UAVid], original copyright [2020].

In the UAVid dataset [16], the shooting scene is urban; the camera angle is about 45 degrees vertical, and the flight height is about 50 metres above the ground. The image resolution is 3840×2160 and 4096×2160, consisting of red, green and blue bands. There are 270 images in the dataset, labelled with eight categories, which are building, road, static car (s car), tree, low vegetation (low veg), human, moving car (m car) and background clutter (clutter). To fully utilize the image data, the images and labels were manually cropped in chunks to 960×720 pixels to obtain 3240 samples, divided into training and validation sets by a 9:1 ratio. In order to facilitate subsequent network training, the size of each sample was uniformly adjusted to 512×512 pixels in the data preparing step before training. As shown in Fig 4, the first row shows the cropped original images, and the second row shows the corresponding labels.

**Fig 5:**
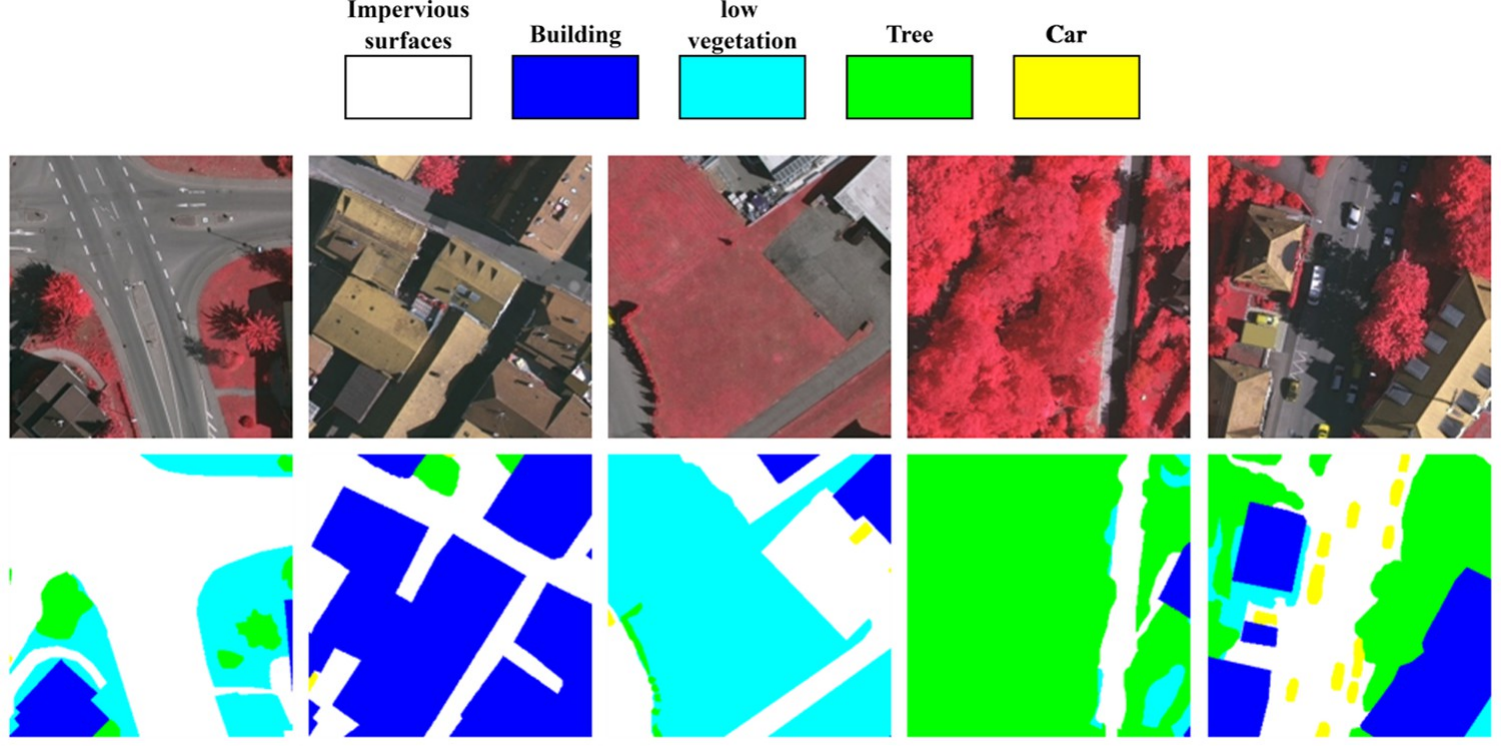
ISPRS Vaihingen dataset example. Reprinted from [17] under a CC BY license, with permission from [DGPF], original copyright [2010].

The Vaihingen dataset [17] used in this paper were provided by Working Group (WG) III/4 of ISPRS from the Vaihingen area of Germany in the context of the “ISPRS test project on urban classification and 3D building reconstruction”. Vaihingen dataset contains 33 remotely sensed images extracted from a larger top-level orthophoto. There are 6 categories: impervious surfaces, buildings, low vegetation, trees, cars, and clutter. The images are 8-bit TIFF files with a resolution of 0.09 m for the ground sample. The three bands of the TIFF files correspond to the near infrared, red and green bands delivered by the camera. The images varied in pixel size with an average size of 2494 × 2064. To enhance the data and adapt to the hardware environment, we cropped the images with overlap: width overlap 370 and height overlap 320. Each image and label were manually cropped to a size of 512×512 pixels to obtain 3269 samples, with a training set and validation set ratio of 9:1. As shown in **Fig 5**, the first row shows the cropped original image, and the second row shows the corresponding labels.

### 4.2. Experimental settings

We perform the experiments on a desktop running Ubuntu 18.04 with 2.50GHZ Intel Xeon E5-2678 CPU, 32 GB Memory, and an NVIDIA 1080Ti Graphics Card. The experiments were run based on PyTorch 1.6. In the training course, we choose stochastic gradient descent (SGD) as the optimizer, and set momentum and weight decay factor to 0.9 and 0.0004 respectively. In addition, based on the results of comparison experiments, we set the initial learning rate and batch size to 0.03 and 6 respectively.

### 4.3. Experiment analysis

#### 4.3.1. Effectiveness analysis of EMNet

To verify the validity of EMNet, Deeplabv3+_Xception [8] (Dv_Xtion), Deeplabv3+_MobileNetV2 (Dv_Mnetv2) and BiSeNetV2 [14] models were selected for experimental comparative analysis.

**Fig 6:**
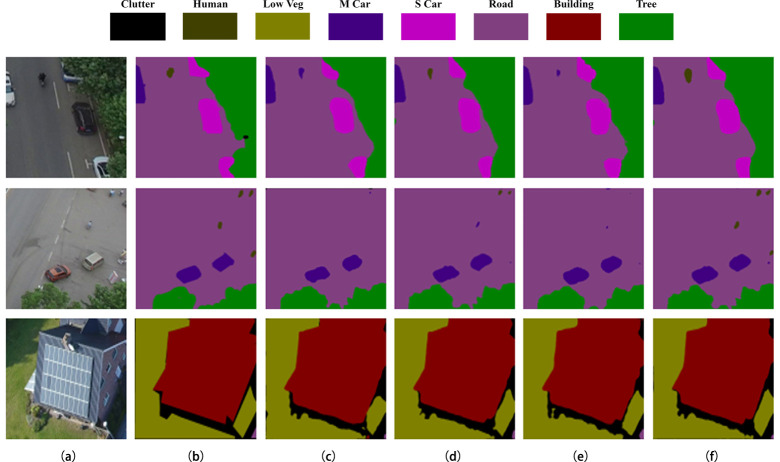
Comparison of segmentation results on UAVid dataset. (a) The original image; (b) The label; (c) The segmentation result of Dv_Xtion; (d) The segmentation result of Dv_Mnetv2; (e) The segmentation result of BiSeNetV2; (f) The segmentation result of EMNet. Reprinted from [16] under a CC BY license, with permission from [UAVid], original copyright [2020].

From Fig 6, it can be seen that the segmentation accuracies of Dv_Xtion and Dv_Mnetv2 are relatively low, as they lack edge information and ignore the target location of small targets and the recovery of edge information. The same problem exists with the BiSeNetV2, as it fails to recognize the “human” object, and its predicted segmentation boundaries of “s car” and “low veg” are not precise enough. In contrast, EMNet addresses the above problems by adding an EDM module and MultiL structure to make the model more applicable to high-resolution UAV remote sensing images.

Table 3 shows the mIoU and mPA values of different models on the UAVid test set. Table 3 also presents the number of parameters (Parameters) and floating point operations (FLOPs) of each model, and these two statistics are independent of the dataset. We can see that EMNet outperforms Dv_Xtion, Dv_Mnetv2 and BiSeNetV2 in both mIoU and mPA. Moreover, EMNet is superior to Dv_Xtion in terms of Parameters and FLOPs. EMNet has slightly more parameters than Dv_Mnetv2 and larger FLOPs than Dv_Mnetv2 and BiSeNetV2, which is due to the fact that EMNet is based on multi-task learning to perform both the task of edge detection and semantic segmentation. Considering these evaluation metrics collectively, we can see that EMNet achieves a good balance between computational efficiency and segmentation accuracy.

**Table 3:** Segmentation performance of different models on the UAVid test set.

Model	mIoU(%)	mPA(%)	Parameters/M	FLOPs/GFLOPs
Dv_Xtion	64.35	73.53	55M	83.11
Dv_Mnetv2	68.95	78.37	**5.8 M**	26.49
BiSeNetV2	62.14	71.36	14M	**19.06**
EMNet (our)	**71.46**	**80.46**	6M	39.34

Note: Bold numbers are the best results.

As shown in Table 4 that EMNet has the highest IoU in all categories, especially on small target segmentation, such as “human”. The above experimental results show that EMNet has notable segmentation performance on UAV remote sensing images. We also performed a t-test (p = 5.23×10^−5^ < 0.05), which indicates that our method significantly outperforms all baseline methods.

**Table 4:** mIoU values for each model for different categories on the UAVid test set.

Model	Class IoU(%)								mIoU(%)
	Clutter	Building	Road	Low Veg	Tree	M Car	Human	S Car	
Dv_Xtion	67.76	90.87	80.82	59.66	79.13	69.86	6.53	60.16	64.35
Dv_Mnetv2	70.34	91.19	82.37	63.54	79.52	71.31	27.17	66.16	68.95
BiSeNetV2	67.29	89.75	81.02	55.79	76.34	66.92	0.23	59.76	62.14
EMNet (our)	**73.27**	**92.03**	**85.13**	**64.73**	**81.05**	**73.32**	**35.12**	**67.01**	**71.46**

Note: Bold numbers are the best results.

**Fig 7:**
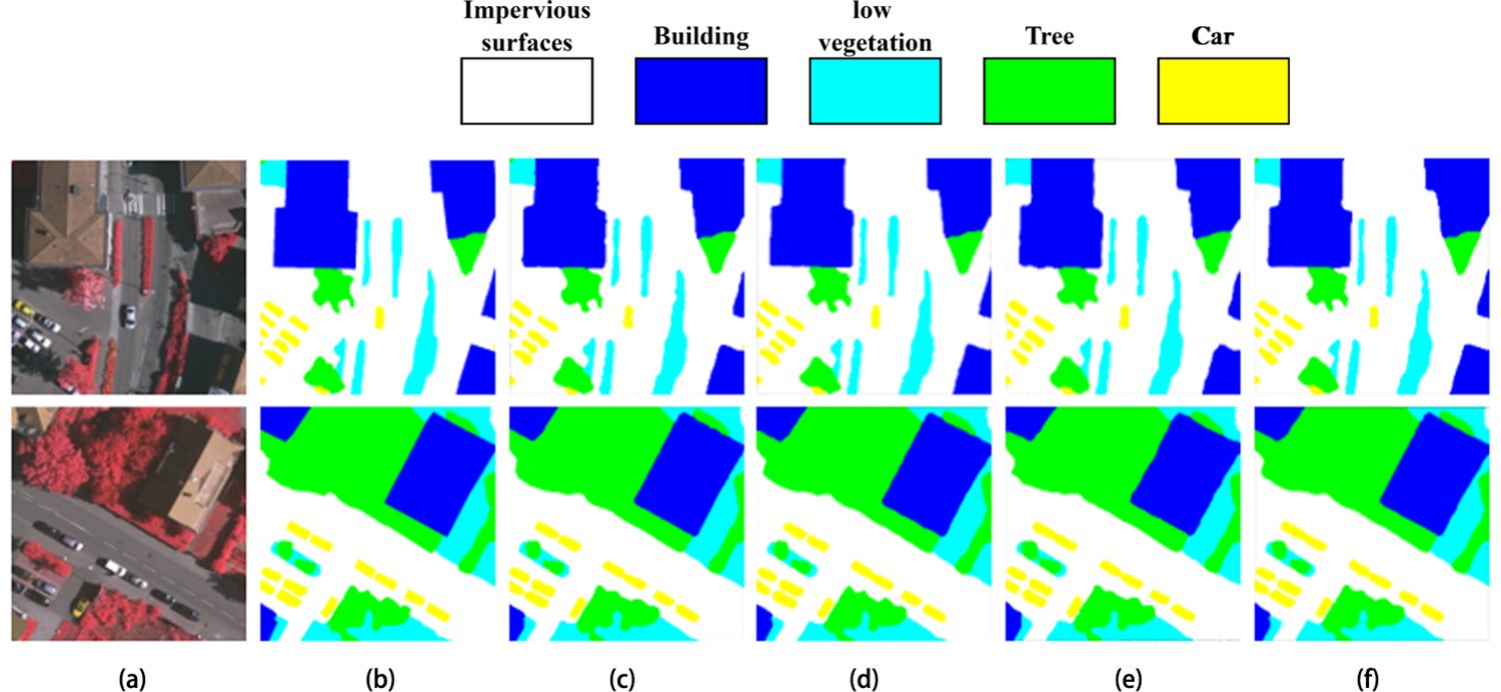
Comparison of segmentation results on ISPRS Vaihingen dataset. (a) The original image; (b) The label; (c) The segmentation result of Dv_Xtion; (d) The segmentation result of Dv_Mnetv2; (e) The segmentation result of BiSeNetV2; (f) The segmentation result of EMNet. Reprinted from [17] under a CC BY license, with permission from [DGPF], original copyright [2010].

To further verify the validity of EMNet, experiments were also conducted on the publicly available dataset ISPRS Vaihingen, the comparison in Fig 7 also shows that the segmentation accuracy of EMNet is higher than other models.

From Table 5, we can see that the evaluation metrics of both mIoU and mPA of EMNet outperformed Dv_Xtion, Dv_Mnetv2, and BiSeNetV2.

**Table 5:** Segmentation performance of different models on the ISPRS Vaihingen test set.

Model	mIoU(%)	mPA(%)
Dv_Xtion	91.28	95.20
Dv_Mnetv2	91.34	95.13
BiSeNetV2	90.37	94.75
EMNet (our)	**91.80**	**95.42**

Note: Bold numbers are the best results.

#### 4.3.2. Ablation analysis of EDM and MultiL modules

**Fig 8:**
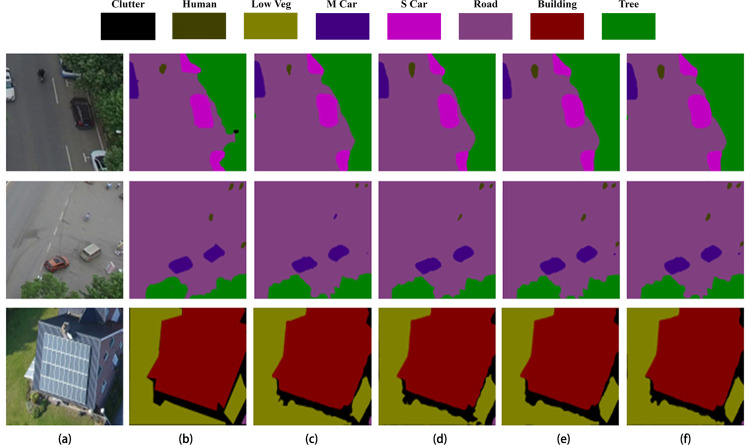
Comparison of segmentation results in ablation experiments. (a) The original image; (b) The label; (c) The segmentation result of Dv_Mnetv2; (d) The segmentation result of Dv_Mnetv2+EDM; (e) The segmentation result of Dv_Mnetv2+MultiL; (f) The segmentation result of EMNet. Reprinted from [16] under a CC BY license, with permission from [UAVid], original copyright [2020].

Ablation experiments were conducted to verify the effectiveness of EDM module and MultiL structure in EMNet. Under the same experimental conditions, we regard Deeplabv3+ using MobileNetV2 backbone feature extraction network (Dv_Mnetv2) as the baseline. The segmentation results in ablation experiments are shown in Fig 8.

As can be seen in Fig 8(C), the baseline network fails to identify all the “human” in the original image accurately, while both Dv_Mnetv2+EDM and Dv_Mnetv2+MultiL show improved segmentation results. EMNet combines the advantages of EDM and MultiL has superior performance in image segmentation.

As shown in Table 6, compared with the baseline, the model incorporating an EDM (Dv_Mnetv2+EDM) improved the mIoU and mPA on the test set by 1.15% and 1.57%, respectively; meanwhile, the model containing the MultiL structure (Dv_Mnetv2+MultiL) improved the mIoU and mPA on the test set by 0.33% and 1.08%, respectively.

**Table 6:** Ablation analysis of EDM and MultiL on UAVid test set.

Model	mIoU(%)	mPA(%)
Dv_Mnetv2	68.95	78.37
Dv_Mnetv2+EDM	70.10	79.94
Dv_Mnetv2+MultiL	69.28	79.45
EMNet (Dv_Mnetv2+EDM+MultiL)	**71.46**	**80.46**

Note: Bold numbers are the best results.

## 5. Conclusions

Based on DeepLabv3+, the proposed EMNet model uses the edge detection branch in the encoder to extract edge features and provide edge information for semantic segmentation. A multi-level upsampling method is designed in the decoder to retain the target’s location and boundary information when recovering the feature map. Compared to DeepLabv3+, EMnet is more accurate in identifying small-sized targets and segmenting edges. The experimental results show that the mIoU and mPA of EMNet are 71.46% and 80.46% on dataset UAVid, and 91.80% and 95.42% on the dataset ISPRS Vaihingen. EMNet outperforms other baseline models on all of these metrics and can better perform the semantic segmentation task of UAV remote sensing images.
